# The potential of sodium-glucose cotransporter 2 inhibitors for the treatment of systemic right ventricular failure in adults with congenital heart disease

**DOI:** 10.3389/fcvm.2023.1093201

**Published:** 2023-06-26

**Authors:** Ralph M. L. Neijenhuis, Marieke Nederend, Monique R. M. Jongbloed, Philippine Kiès, Joris I. Rotmans, Hubert W. Vliegen, J. Wouter Jukema, Anastasia D. Egorova

**Affiliations:** ^1^ CAHAL, Center for Congenital Heart Disease Amsterdam Leiden, Leiden University Medical Center, Leiden, Netherlands; ^2^ Department of Cardiology, Leiden University Medical Center, Leiden, Netherlands; ^3^ Department of Anatomy & Embryology, Leiden University Medical Center, Leiden, Netherlands; ^4^ Department of Internal Medicine & Nephrology, Leiden University Medical Center, Leiden, Netherlands; ^5^ Netherlands Heart Institute, Utrecht, Netherlands

**Keywords:** congenital heart disease, adult congenital heart disease (ACHD), heart failure, systemic right ventricle, sodium glucose co-transport-2 (SGLT2) inhibitors, transposition of the great arteries (TGA), congenitally corrected transpoition of the great arteries

## Abstract

**Aims:**

Given the compelling evidence on the effectiveness of sodium-glucose cotransporter 2 inhibitors (SGLT2i) in the conventional heart failure population, SGLT2i deserve exploration in systemic right ventricular (sRV) failure. The initial experience with dapagliflozin in sRV failure patients is described, with a focus on tolerability and short-term effects on clinical outcomes.

**Methods and results:**

Ten patients (70% female, median age 50 years [46.5–52]) with symptomatic sRV failure who received dapagliflozin 10 mg per day on top of optimal medical therapy between 04–2021 and 01–2023 were included. Within 4 weeks, no significant changes in blood pressure, electrolytes, or serum glucose occurred. Creatinine and estimated glomerular filtration rate (eGFR) showed a slight decline (88 ± 17 to 97 ± 23 µmol/L, *p* = 0.036, and 72 ± 14 vs. 66 ± 16 ml/min/1.73m^2^, *p* = 0.020, respectively). At 6 months follow-up (*n* = 8), median NT-proBNP decreased significantly from 736.6 [589.3–1193.3] to 531.6 [400.8–1018] ng/L (*p* = 0.012). Creatinine and eGFR recovered to baseline levels. There were no significant changes in echocardiographic systolic sRV or left ventricular function. New York Heart Association class improved significantly in 4 out of 8 patients (*p* = 0.046), who also showed an improvement in the 6-minute walk test or bicycle exercise test performance. One female patient developed an uncomplicated urinary tract infection. No patients discontinued treatment.

**Conclusion:**

Dapagliflozin was well-tolerated in this small cohort of sRV failure patients. While the early results on the reduction of NT-proBNP and clinical outcome parameters are encouraging, large-scale prospective studies are warranted to thoroughly evaluate the effects of SGLT2i in the growing sRV failure population.

## Introduction

With the improved survival of congenital heart disease patients, clinicians are frequently confronted with heart failure in this heterogeneous population. Complex congenital heart disease with a systemic right ventricle (sRV) forms a distinct entity where the morphological RV functions in the subaortic position and supports the systemic circulation. This occurs in the biventricular setting of transposition of the great arteries (TGA) after the atrial switch procedure according to Mustard or Senning, or in congenitally corrected TGA (ccTGA). Failure of the sRV is a frequent long-term complication in these patients; at 45 years of age, over 50% of TGA patients who underwent the atrial switch and up to 65% of ccTGA patients will have developed symptomatic heart failure (1, 2). Robust evidence for the use of heart failure medication in this group is lacking, and the mechanisms and treatment of sRV failure have been identified as major gaps of knowledge in the latest European and American adult congenital heart disease guidelines (3, 4).

Recently, sacubitril/valsartan (an angiotensin receptor-neprilysin inhibitor, ARNI) has been linked to reduction of N-terminal pro-B-type natriuretic peptide (NT-proBNP) levels, sRV remodeling with improvement of systolic ventricular function, improvements in 6-minute walk test (6MWT) distance, New York Heart Association (NYHA) class, and quality of life in patients with sRV failure (5, 6). These results suggest that heart failure in sRV patients may indeed be amendable to timely pharmacological intervention.

Sodium-glucose cotransporter 2 inhibitors (SGLT2i) are a relatively novel class of drugs that have quickly become a pillar in the treatment of “conventional” left ventricular heart failure, demonstrating a reduced risk of worsening heart failure, hospitalization, and cardiovascular-related death (7). Given the unmet need for evidence-based pharmacological strategies in sRV failure and the compelling evidence on the effectiveness of SGLT2i in the “conventional” heart failure population, SGLT2i have been prescribed to some patients with progressive sRV failure and insufficient response to classical pharmacological heart failure therapy, as demonstrated by Egorova et al. (2022) (8).

In this brief report, we describe the tolerability and short-term neurohumoral, echocardiographic, and clinical effects of dapagliflozin in the treatment of sRV patients with heart failure. Moreover, we discuss the rationale for and propose a future prospective evaluation of SGLT2i effects in this patient group.

## Materials and methods

### Participants

Adult patients with symptomatic sRV failure who were started on dapagliflozin 10 mg p.o. q.d. on top of current optimal medical therapy as part of compassionate care between April 2021 and January 2023 were reviewed for inclusion.

Inclusion criteria were: adults with an sRV in a biventricular circulation with underlying anatomy of TGA after the atrial switch procedure or ccTGA, progressive symptomatic heart failure with NYHA class ≥ II, and at least moderately reduced systolic sRV function as assessed by echocardiography. Progressive symptomatic heart failure was defined as progressive symptoms attributed to heart failure and/or a deterioration in exercise performance as assessed by bicycle exercise test, accompanied by an increase in NT-proBNP levels despite the pharmacological treatment.

### Study design

All patients were followed up in the outpatient clinic according to the smart technology-supported care pathway at the Leiden University Medical Center, as previously described (5). After baseline measurements, patients had an initial evaluation after 2 to 4 weeks and a structured outpatient clinic follow-up visit at 6 months.

Tolerability was evaluated by blood pressure and laboratory investigations (including creatinine, estimated glomerular filtration rate (eGFR), non-fasting plasma glucose, and electrolytes) after 2 to 4 weeks, discontinuation of treatment, and the occurrence of side effects or adverse events.

At the structured 6 months follow-up visit, the biomarker NT-proBNP was evaluated as a surrogate for neurohumoral activation status and heart failure-related prognosis. Furthermore, hemoglobin, hematocrit, electrolytes, creatinine, eGFR, aspartate transaminase (AST), alanine transaminase (ALT), gamma-glutamyltransferase (GGT), HbA1c, non-fasting serum glucose, and uric acid changes were evaluated. Systolic sRV and left ventricular (LV) function were assessed by transthoracic echocardiography as previously described (5, 9). Changes in functional outcomes were assessed by NYHA functional class, 6MWT performance, and bicycle ergometry with VO_2_ max.

### Ethics approval statement

All tests and procedures performed involving human participants were in accordance with the ethical standards of the institutional and/or national research committee and with the 2013 Helsinki declaration or comparable ethical standards. Appropriate local scientific board approval was obtained and the need for written informed consent was waived by the institutional medical ethical board (protocol N22.120). All patients provided consent for registration of their data and publication.

### Statistical analysis

All statistical analyses were performed in SPSS version 25 (IBM Corp, Armonk, NY). The figure was generated with Graphpad Prism version 9.3.1 (GraphPad Software, San Diego, California USA). Normally distributed continuous data were displayed as mean (± standard deviation) and non-normally distributed continuous data were displayed as median [interquartile range Q1—Q3]. Proportions were displayed as numbers (percentages). Normality was tested with the use of the Shapiro-Wilk test. For the comparison of continuous values between time points, paired samples *t*-tests or Wilcoxon signed-rank tests were used as appropriate. For the comparison of paired ordinal data, the Wilcoxon signed-rank test was used. A *p*-value < 0.05 was considered statistically significant.

## Results

Ten consecutive patients were included in this retrospective cohort. Six (60%) had underlying anatomy of TGA late after the atrial switch, and 4 (40%) had ccTGA. Seven patients (70%) were female and the median age at treatment initiation was 50 years [46.5–52]. Baseline patient characteristics are shown in Table 1. All patients were on at least one heart failure drug, including beta-blocker, ARNI, mineralocorticoid receptor antagonists, or diuretics before starting dapagliflozin. Nine patients (90%) were on a maximum tolerated dose of an ARNI, and 70% were on diuretics before starting dapagliflozin.

**Table 1:** Baseline patient characteristics.

Baseline (*n* = 10)	
Age, years [IQR1-IQR3]	50 [46.5–52]
Female (%)	7 (70)
Weight, kg (± SD)	73.9 ± 17.5
Body Mass Index, kg/m² (± SD)	24.2 ± 4.0
*Anatomy (%)*
TGA atrial switch	6 (60)
ccTGA	4 (40)
*Concomitant defects (%)*
ccTGA + VSD	1 (10)
ccTGA + VSD + monoatrium	1 (10)
TGA + VSD + PS	1 (10)
ccTGA + VSD + Ebstein-like TV	1 (10)
TGA + VSD + ASD + PS + PAPVD + persistent SVC	1 (10)
None	5 (50)
Number of cardiac surgeries [IQR1-IQR3]	1.5 [1–2]
*Atrial switch procedure (%)*
Mustard	4 (40)
Senning	2 (20)
*History of TV surgery (%)*
Mechanical TV	3 (30)
TV annuloplasty	2 (20)
History of atrial arrhythmia (%)	9 (90)
History of atrial ablation procedure (%)	6 (60)
Type II diabetes mellitus (on oral treatment) (%)	1 (10)
*NYHA functional class (%)*
Class II	4 (40)
Class III	4 (40)
Class IV	2 (20)
Oxygen saturation at ambient air, % [IQR1-IQR3]	99 [98–99]
Systolic blood pressure, mmHg (± SD)	107 ± 17
Diastolic blood pressure, mmHg (± SD)	67 ± 9
eGFR, ml/min/1.73m^2^ (± SD)	72 ± 14
Patients with a heart failure-related hospitalization in past year (%)	1 (10)
Pharmacological therapy (%)	
Beta blocker	5 (50)
ACE-i/ARB/ARNI	0/0/9 (90)
MRA	6 (60)
Diuretics (loop and/or thiazide)	7 (70)
Antiarrhythmic drugs	3 (30)
Cardiac Implantable Electronic Devices (%)	
AAIR-pacemaker	1 (10)
DDD-pacemaker	1 (10)
DDD-ICD	4 (40)
CRT-D	2 (20)
Baseline exercise performance	
6MWT distance, m [IQR1-IQR3]	604 [501–645]
VO_2_ max, mL/kg/min (± SD)	15.2 ± 4.0
%-pred. VO_2_ max (± SD)	54 ± 14
Baseline echocardiography	
*sRV function (%)*	
Moderately reduced	6 (60)
Severely reduced	4 (40)
sRV GLS, % (± SD)	−10.5 ± 3.3
sRV FAC, % (± SD)	19.1 ± 6.0
TAPSE, mm (± SD)	12 ± 2
*Tricuspid regurgitation, grade (%)*
Grade I or less	3 (30)^$^
Grade II	7 (70)
*Subpulmonary LV function (%)*
Good	8 (80)
Mildly reduced	2 (20)

*Proportions are presented as n (%). Values are presented as mean (± standard deviation) or median [IQR1-IQR3] as appropriate.* 6MWT, 6-minute walk test; ACE-i, angiotensin-converting enzyme inhibitor; ARB, angiotensin receptor blocker; ARNI, angiotensin receptor-neprilysin inhibitor; ASD, atrial septal defect; ccTGA, congenitally corrected transposition of the great arteries; CRT, cardiac resynchronization therapy; eGFR, estimated glomerular filtration rate; FAC, fractional area change; GLS, global longitudinal strain; ICD, implantable cardioverter defibrillator; LV, left ventricle; MRA, mineralocorticoid receptor antagonist; NYHA, New York Heart Association; PAPVD, partial anomalous pulmonary venous drainage; PS, pulmonary stenosis; sRV, systemic right ventricle; SVC, superior vena cava; TAPSE, tricuspid annular plane systolic excursion; TGA, transposition of the great arteries; TV, tricuspid valve; VSD, ventricular septal defect.

^$^ The three patients with grade I or less tricuspid regurgitation at baseline all had a mechanical TV.

At baseline, 4 patients had severely reduced and 6 had moderately reduced systolic sRV function. Three patients had a mechanical tricuspid valve and the other 7 patients had grade II tricuspid regurgitation. The systolic subpulmonary LV function was good in 8 and mildly reduced in 2 patients.

### Tolerability

After 2–4 weeks of treatment with dapagliflozin, no statistically significant changes in systolic blood pressure (107 ± 17 to 103 ± 16 mmHg, *p* = 0.509), diastolic blood pressure (67 ± 9 to 63 ± 10 mmHg, *p* = 0.406), non-fasting plasma glucose levels (4.9 [4.4–6.4] to 5.0 [4.3–7.1] mmol/L, *p* = 0.889), or electrolytes were observed. Creatinine increased (88 ± 17 to 97 ± 23 µmol/L, *p* = 0.036) and eGFR decreased (72 ± 14 vs. 66 ± 16 ml/min/1.73m^2^, *p* = 0.020) significantly. A 60-year-old female patient developed a urinary tract infection which was treated with oral antibiotics. No other side effects or adverse events were observed and none of the patients discontinued the treatment.

One heart failure-related hospitalization occurred during follow-up; patient 1 had a short admission (<24 h) after 4 months with mild congestion which resolved after a single intravenous administration of loop diuretics. Additionally, this patient required escalation in the dose of spironolactone and bumetanide despite treatment with dapagliflozin and was listed for heart transplantation. In the 6 months preceding SGLT2i therapy, this patient experienced 4 heart failure-related admissions. The dose of furosemide was reduced in patient 2 and triamterene could be stopped in patient 3. No concurrent changes in medical therapy occurred in the other 7 patients during follow-up.

### Short-term effects of SGLT2i

**Figure 1:**
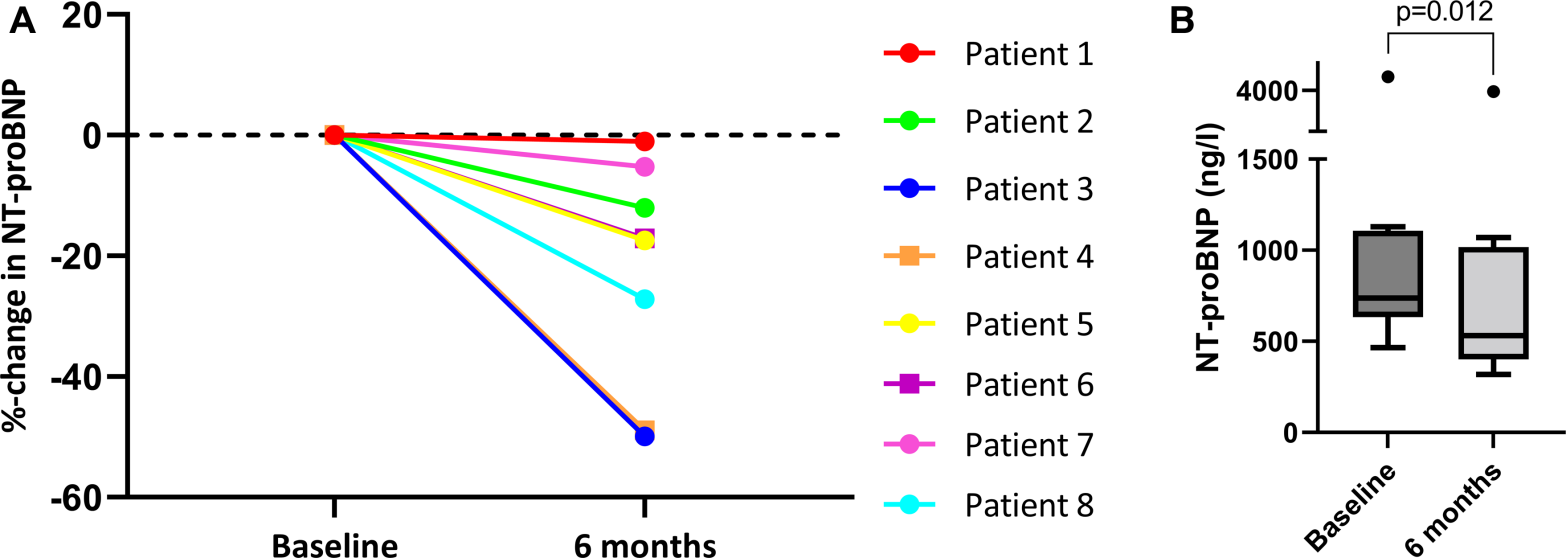
Changes in NT-proBNP from baseline to 6 months for patients 1–8. (**A**) Percentage change in NT-proBNP from baseline to 6 months follow-up for individual patients 1 to 8. (**B**) Box-and-whiskers plot presenting the absolute NT-proBNP (ng/L) levels at baseline and 6 months follow-up for patients 1 to 8. The *p*-value refers to the comparison of the median values between baseline and 6 months follow-up (Wilcoxon signed-rank test). *NT-proBNP, N-terminal pro-B-type natriuretic peptide*.

Eight patients (80%) had a 6 months follow-up visit. From baseline to 6 months, median NT-proBNP decreased significantly from 736.6 [589.3–1,193.3] to 531.6 [400.8–1,018] ng/L (*p* = 0.012) (Figure 1). Creatinine (85 [74–100] to 77 [67–108] µmol/L, *p* = 0.575) and eGFR (71 ± 14 to 71 ± 19 ml/min/1.73m^2^, *p* = 0.539) recovered after the initial deterioration in renal function, with no significant change from baseline to 6 months (Table 2). There were no significant changes in body weight (73.9 ± 17.5 to 71.0 ± 16.9 kg, *p* = 0.957) or body mass index (mean 24.2 ± 4.0 to 24.1 ± 4.3 kg/m^2^, *p* = 0.908). There were no statistically significant changes in systolic sRV or LV function and dimensions as assessed with transthoracic echocardiography from baseline to 6 months follow-up (Table 3). Changes in functional outcome parameters from baseline to 6 months are shown in Table 4. A significant improvement in NYHA class was observed in 4 out of 8 patients (*p* = 0.046), and no patients experienced a clinical deterioration. These 4 patients also showed an improvement in either 6MWT distance or exercise test performance, although these changes were not statistically significant.

**Table 2:** Changes in laboratory parameters from baseline to 6 months for patients 1–8.

	Baseline	6 months	*p*-value
NT-proBNP, ng/L [IQR1-IQR3]	736.6 [589.3–1,193.3]	531.6 [400.8–1,018]	*0*.*012*
Hemoglobin, mmol/L (± SD)	8.5 ± 1.4	8.3 ± 0.8	0.635
Hematocrit, L/L (± SD)	0.416 ± 0.063	0.408 ± 0.032	0.634
Sodium, mmol/L (± SD)	140 ± 3	139 ± 2	*0*.*044*
Potassium, mmol/L (± SD)	4.2 ± 0.3	4.3 ± 0.7	0.441
Creatinine, µmol/L [IQR1-IQR3]	85 [74–100]	77 [67–108]	0.575
eGFR, mL/min/1.73m2 (± SD)	71 ± 14	71 ± 19	0.539
AST, U/L [IQR1-IQR3]	29 [23–38]	30 [22–38]	0.799
ALT, U/L [IQR1-IQR3]	30 [22–66]	30 [22–37]	0.401
GGT, U/L [IQR1-IQR3]	50 [28–173]	53 [31–93]	0.138
HbA1c, mmol/mol [IQR1-IQR3]	41.9 [38.3–44.0]	42.3 [37.9–42.9]	0.128
Serum glucose (non-fasted), mmol/L [IQR1-IQR3]	4.9 [4.4–6.4]	5.1 [4.6–6.2]	0.833
Uric acid, mmol/L (± SD)	0.45 ± 0.18	0.32 ± 0.14	0.088

*Changes in laboratory parameters from baseline to 6 months follow-up. Values are presented as mean (± standard deviation) or median [IQR1-IQR3]. Paired t-test or Wilcoxon signed-rank tests are performed as appropriate*. AST, aspartate transaminase; ALT, alanine transaminase; eGFR, estimated glomerular filtration rate; GGT, gamma-glutamyltransferase; NT-proBNP, N-terminal pro-B-type natriuretic peptide.

**Table 3:** Echocardiographic changes from baseline to 6 months for patients 1–8.

	Baseline	6 months	*p*-value
sRV GLS, % [IQR1-IQR3]	−11.2 [−12.8—−8.2]	−11.4 [−12.5—−10.2]	0.889
sRV FAC, % (± SD)	19.1 ± 6.0	19.0 ± 7.1	0.707
sRV s’ [IQR1-IQR3]	4.9 [4.4–6.0]	5.5 [4.0–6.5]	0.665
TAPSE, mm (± SD)	12.0 ± 1.9	12.6 ± 1.3	0.325
sRV EDD, mm (± SD)	55.0 ± 5.7	55.8 ± 6.3	0.897
Tricuspid regurgitation, grade [IQR1-IQR3]	2 [1–2]	2 [1–2]	1.000
LV GLS, % (± SD)	−22.4 ± 7.0	−20.9 ± 3.8	0.482
MAPSE, mm [IQR1-IQR3]	18 [17–22.5]	19.7 [16.5–22.8]	0.611
Mitral regurgitation, grade [IQR1-IQR3]	1 [1–2]	1 [1–2]	1.000

*Changes in echocardiographic parameters from baseline to 6 months follow-up. Values are presented as mean (± standard deviation) or median [IQR1-IQR3]. Overall changes are not statistically significant, as assessed with paired t-test or Wilcoxon-signed-rank test as appropriate*. FAC, fractional area change; GLS, global longitudinal strain; LV, left ventricle; MAPSE, mitral annular plane systolic excursion; sRV, systemic right ventricle; EDD, end-diastolic diameter; TAPSE, tricuspid annular plane systolic excursion.

**Table 4:** Changes in NYHA class and exercise capacity from baseline to 6 months for patients 1–8.

Patient	NYHA class at baseline	NYHA class at 6 months *(p = 0.046)*	*Δ* 6MWT (m) *(p = 0.345)*	*Δ* VO_2_ max (mL/kg/min) *(p = 0.499)*	*Δ* %-pred. VO_2_ max *(p = 0.386)*	*Δ* exercise capacity (W) *(p = 0.793)*	*Δ* %-pred. exercise capacity *(p = 0.723)*
**1**	IV	III	NA	+ 6.1	+23	+43	+28
**2**	III	II	+34	−0.9	−1	+0	+0
**3**	III	II	+75	+2.1	+10	+20	+16
**4**	IV	III	−82	+1.2	+4	+1	+3
**5**	III	III	−65	−3.0	−13	−20	−19
**6**	III	III	NA	−0.3	+3	+1	+2
**7**	II	II	−129	0	+3	−28	−16
**8**	II	II	−7	+0.4	−2	0	+2

Absolute change from baseline to 6 months follow-up (*Δ*). Changes are not statistically significant, as is indicated by the *p*-values and assessed with paired t-test or Wilcoxon-signed-rank test as appropriate. NYHA, New York Heart Association; 6MWT, 6 min walking test.

## Discussion

Dapagliflozin was well-tolerated in this cohort of 10 patients with symptomatic sRV failure: (1) blood pressure, plasma glucose, and electrolytes remained stable while renal function recovered to baseline levels after an initial dip, (2) no patients discontinued treatment, (3) no relevant side effects were observed, and (4) no unexpected deterioration of heart failure was observed.

In the 8 patients with a structured 6 months follow-up visit, we observed a significant median NT-proBNP reduction of 17.3% [−43.5–−6.9]. To compare, a 45% reduction in NT-proBNP levels was documented at 6 months in sRV failure patients treated with sacubitril/valsartan (5). While the reduction in NT-proBNP was greater in this larger cohort of sRV failure patients on sacubitril/valsartan, 9 out of 10 patients in the current study had progressive heart failure despite treatment with sacubitril/valsartan in maximally tolerated dose, suggesting that SGLT2i might be of additional value “on top of” sacubitril/valsartan. The one patient who was not on sacubitril/valsartan at baseline did not tolerate it previously. Sacubitril/valsartan was not recently stopped (<6 months) and also not started during the follow-up of the current study period.

A comparable magnitude of NT-proBNP reduction was documented in the large randomized controlled trials investigating SGLT2i in conventional left ventricular heart failure with reduced ejection fraction. In the DAPA-HF and EMPEROR-reduced trials, median NT-proBNP reductions of 13.7% and 12.9%, respectively were reported (10, 11). Whether this similar reduction in NT-proBNP levels will also be associated with a comparable improvement in clinical outcomes and mortality remains to be investigated.

NT-proBNP is released by both the ventricular and atrial myocytes as a response to elevated cardiac wall stress, and has prognostic value in sRV failure patients specifically; a decrease in NT-proBNP has been correlated with improved clinical status, reduced risk of hospitalization, and lower mortality in sRV failure patients (12). Elevated NT-proBNP levels may thus indicate the presence of pressure and/or volume overload associated with heart failure in sRV patients (13). The actual mechanisms of SGLT2i contributing to the beneficial effects observed in the general heart failure population are not yet fully understood. Potential mechanisms that could target the elevated wall stress and thus contribute to the decrease in NT-proBNP levels we observed are; (1) blood pressure lowering and stimulation of natriuresis and diuresis, thereby lowering cardiac afterload, (2) inhibition of sympathetic nervous system activation, and (3) improvement of vascular smooth muscle and endothelial function, decreasing vascular resistance and inducing direct vasorelaxation (14).

Regarding the short-term outcomes, a significant improvement in NYHA class was observed. Nevertheless, there were no significant changes in any of the exercise parameters nor in the echocardiographic parameters assessing systolic ventricular function. This might be attributed to the limited cohort, small effect size, or the variability in the echocardiographic assessment of sRV function.

Another potential advantage of SGLT2i is the practical ease of implementation and adherence when compared to other heart failure drugs, as the target dose and starting dose are identical and can be implemented over a wide range of renal functions. When comparing SGLT2i to other drugs with more profound anti-hypertensive effects like ARNIs or beta blockers, SGLT2i might prove to be especially beneficial in relatively young adult congenital heart disease patients, in whom hypotension rather than hypertension usually is a limiting factor.

The initial results seem encouraging and demonstrate that SGLT2i treatment is feasible and safe, even in advanced sRV failure patients. Although no firm conclusions can be drawn regarding the clinical effects, the current data is promising and worthy of further validation in a larger prospective setting, particularly when considering the growing unmet need for adequate heart failure treatment in the adult congenital heart disease population. Therefore, we call for international collaboration and the initiation of a prospective trial to study the effects of SGLT2i as a promising treatment option for sRV failure.

## Data Availability

The original contributions presented in the study are included in the article. Requests to access these datasets should be directed to Anastasia Egorova, a.egorova@lumc.nl
